# Characterizing the Flow of Thickened Barium and Non-barium Liquid Recipes Using the IDDSI Flow Test

**DOI:** 10.1007/s00455-018-9915-6

**Published:** 2018-06-11

**Authors:** Carly E. A. Barbon, Catriona M. Steele

**Affiliations:** 1 Swallowing Rehabilitation Research Laboratory, Toronto Rehabilitation Institute—University Health Network, 550 University Avenue, 12th floor, Toronto, ON M5G 2A2 Canada; 2 Department of Speech-Language Pathology, Faculty of Medicine, Rehabilitation Sciences Institute, University of Toronto, Toronto, ON Canada; 3 Board of Directors, International Dysphagia Diet Standardisation Initiative, Brisbane, Australia

**Keywords:** Keyword, Deglutition, Deglutition disorders, Dysphagia, Texture modification, Videofluoroscopy, Thickened liquids, Barium

## Abstract

The use of thickened liquids for dysphagia management has become wide-spread. Videofluoroscopy is commonly used to determine dysphagia severity and to evaluate the effectiveness of interventions, including texture modification, but this requires the use of radio-opaque contrast media. In order for the results of a videofluoroscopy to have validity with respect to confirming swallowing safety and efficiency on different liquid consistencies, it is important to understand the flow characteristics of the contrast media used and how the flow of these stimuli compares to the flow of liquids that are provided outside the assessment context. In this study, we explored the flow characteristics of 20% w/v barium and non-barium stimuli prepared using starch and gum thickeners to reach the slightly, mildly and moderately thick liquid categories defined by the International Dysphagia Diet Standardisation Initiative (IDDSI). Our goal was to identify recipes that would produce stimuli with stable flow properties over a 3 h time frame post mixing. Thickener concentration was titrated to achieve matching flow (i.e., IDDSI Flow Test results within a 1 ml range) across the four stimulus types (non-barium starch, non-barium gum, barium starch, barium gum) within each IDDSI level. The combination of barium and thickeners resulted in further thickening, particularly with starch-based thickening agents. A probe of the influence of refrigeration showed no difference in flow measures between chilled and room temperature stimuli over a 3-h time frame. Overall, recipes with stable flow over three hours were identified for all barium and non-barium liquids tested.

## Introduction

Diet texture modification is widely recommended to promote and maintain patient safety when managing dysphagia [1]. Thickened liquids are used as an intervention based on evidence that boluses with higher viscosity travel more slowly through the oropharynx, and are less likely to be aspirated [2]. Despite the widespread use of thickened liquids, little is known regarding the specific flow characteristics that are needed to achieve therapeutic benefit [2]. Recent studies have also shown a risk of greater post-swallow residue with extremely thick liquids [2]. In light of this evidence of potential risk as well as benefit with the use of thickened liquids, the effectiveness and optimal consistency of thickened liquids should be evaluated on a case by case basis using instrumental swallowing examinations.

In order for a bolus to be visualized in videofluoroscopy, a radiographic contrast agent must be used. In North America, barium is the most common contrast agent used. In the United States, there is one line of available barium products that comes in an array of different consistencies, specifically intended for imaging of the oropharynx (Bracco Varibar^®^). However, this line of products is not currently approved for clinical use outside the United States. Consequently, the standard of care in Canada and many other countries is for clinicians to prepare barium stimuli in different consistencies using off-label recipes. However, the addition of a thickening agent to a barium solution (or vice versa, the addition of barium powder to a pre-thickened liquid) may result in further thickening [3]. When this happens, the validity of the assessment stimulus for predicting swallowing function outside the context of the exam becomes a concern [2, 4, 5].

In addition to the amount of thickener that is used in a recipe for a thickened liquid [6, 7], a number of other factors may influence the flow characteristics of the resulting stimulus. These factors have relevance for the preparation of thickened barium stimuli as well as non-barium stimuli. Since the first descriptions of thickened liquids used for managing aspiration risk [8, 9], increasing varieties of thickeners and pre-thickened liquids have emerged on the market. Variations in liquid flow occur across different thickener types (e.g., modified corn starch versus xanthan gum) [10, 11]. Other factors that may be relevant include thickening technique (e.g., hand-mixed versus machine-mixed) [12]; time post mixing [10, 11, 13]; temperature [13]; and characteristics of the liquid being thickened (e.g., pH, fat content, protein content, and volume of liquid in the recipe) [14, 15]. Non-linear concentration curves may be observed with some thickening agents [16].

Historically, guidelines regarding thickened liquids used in dysphagia management have defined categories of progressively thicker liquids in terms of viscosity [17, 18] (National Dysphagia Diet Task Force), most commonly reported at a shear rate of 50/s [17]. However, neither the equipment nor the expertise required to measure viscosity at controlled shear rates is accessible to clinicians. Recently, the International Dysphagia Diet Standardization Initiative (www.iddsi.org) has released new guidelines for classifying liquid thickness according to gravity flow through a syringe [18, 19]. Three levels of thickened liquid (slightly thick, mildly thick, and moderately thick) are defined based on the height of the residual fluid column (in ml) after 10 s of flow through a standard 10 ml slip tip syringe (Becton–Dickinson manufacturer code 301604). A fourth level of extremely thick liquids shows no flow through the syringe; supplementary spoon tilt and fork drip tests are recommended to confirm the characteristics of liquids at this level.

In this article, we explore the flow characteristics of barium stimuli prepared according to recipes using two different commercial thickeners (modified corn starch and xanthan gum) in combination with water and barium sulfate powder, and compare the results of these tests to non-barium stimuli prepared using water and the same thickening agents. The method of flow measurement selected for this study is the gravity-flow test recommended by the International Dysphagia Diet Standardisation Initiative (IDDSI) [18, 19] The primary objective was to identify recipes for barium stimuli that would meet the IDDSI definitions of slightly, mildly, and moderately thick liquids [18] at 1-h post mixing and remain stable within these defined flow ranges up to 3-h post mixing. The process involved testing of different concentrations of thickener to determine the need to adjust the amount of thickener in a barium recipe compared to the amounts used when preparing non-barium stimuli. As an additional objective, we wanted to conduct a preliminary probe of the impact of temperature (i.e., room temperature vs. chilled) on the flow characteristics of thickened barium stimuli.

## Methods

### Stimulus Mixing

Thickened non-barium liquids were prepared using Nestlé ThickenUp^®^ (starch) and Nestlé ThickenUp^®^ Clear^®^ (xanthan-gum) powders and a commercially available lemon-flavored water (Nestlé^®^ Lemon Splash). The non-barium stimuli were developed for use in a related study of swallowing; lemon-flavored water was chosen to make these stimuli more palatable than thickened water. The taste of the unthickened Lemon Splash product was rated by a blinded taste panel, who judged the intensity of the sourness to be similar to a solution of 0.02% lemon juice and sweetness to 0.02% sucrose in water. This degree of sourness falls well below the levels reported to impact swallowing behaviors [20]. For the barium stimuli, the same thickening agents were mixed with a 20% w/v concentration barium suspension, comprising bottled water (Nestlé^®^ Pure Life) and Bracco E-Z-Paque^®^ 96% w/w barium powder. All stimulus mixing was performed by a single research team member using a commercially available Bosch stand mixer (Model MUM4405UC, 4 speed, 400 W motor). Mixer speed was confirmed prior to the experiment by placing markers on the whisk, video recording the whisk in motion, and determining the number of rotations per second. The standard operating procedure for mixing was as follows:

1. The desired amount of water was poured into the Bosch stand mixer mixing bowl, with the amount confirmed by weight on an OHAUS digital balance (model number PA1502 analytical scale: capacity: 1.5 kg; readability 0.01 g).
2. The mixing bowl was removed, and a plastic nonstatic weigh boat (VWR^®^ 20.5 cm^3^ capacity) was placed on the balance.
3. The balance was tared to account for the weigh boat, and the thickener was added until the target weight was achieved.
4. For the barium stimuli, the required amount of barium powder was similarly measured using a weigh boat.
5. The water was set in motion using the Bosch stand mixer at a low spin speed of 60 rpm.
6. The weighed barium powder was added to the water, while in motion.
7. The weighed thickening powder was added, while the water was in motion. This process was completed slowly (over 10–20 s) in order to avoid clumping of the thickener.
8. Once the thickener was added, the speed was increased by one level for 10 s, and then lowered back to low speed.
9. The liquid was then left to mix for a period of 1 min and 50 s (2-min mixing time overall) at a slow spin speed of 60 rpm.
10. The liquid stimulus was portioned into separate, disposable cups with lids to allow for testing at subsequent timepoints.

Trials of various amounts of thickener were tested at the 1-h mark, beginning with the manufacturer instructed amount. If the liquid flow fell outside the targeted IDDSI range, iterative testing of small differences in thickener concentration was explored, as follows:

1. Non-barium thickened with starch in concentrations of 4.1, 4.15, 4.2, 4.75, 4.77, 4.8, 5.0, 5.5, 5.8, 6.0, and 7.8 g/100 ml.
2. Non-barium thickened with xanthan gum in concentrations of 0.65, 1.0, 1.1, 1.25, 1.3, 1.4, and 2.1 g/100 ml.
3. Barium thickened with starch in concentrations of 2.64, 2.85, 2.87, 3.0, 3.3, 3.75, 3.8, 3.9, 5.1, 6.7, 7.4, and 7.6 g/100 ml.
4. Barium thickened with xanthan gum in concentrations of 0.35, 0.5, 0.9, 1.0, 1.02, 1.05, 2.0, 2.2, 4.2, and 4.5 g/100 ml.

### Flow Testing

The IDDSI Flow Test was used to measure the flow properties of all liquid stimuli created for the study. Detailed instructions for this test can be found at: http://iddsi.org/framework/drink-testing-methods/. All tests were conducted by a single research team member. Each stimulus was tested in triplicate at each measurement timepoint. Clean, fresh syringes were used for each sample. Stability in flow was considered to have been achieved when results across successive timepoints remained within a 1 ml flow test result range. In the event that the observed range in flow test results across three repeated samples spanned more than 1 ml, a fourth sample was tested to correct for possible outlier results attributable to the possibility that small lumps or bubbles might have blocked the syringe nozzle. In these cases, the outlier result was discarded, and the three closest test results were retained for analysis. Similarity in flow across the different stimuli (barium + thickener combinations) was considered to have been achieved when results at the same timepoint fell within a flow test result range of 1 ml across stimuli.

### Stability Testing

To assess stability of the flow characteristics of the stimuli, we performed repeated testing over different timepoints (1-, 2-, and 3-h post mixing), with a goal to identify recipes that produced liquids with IDDSI Flow Test results that remained stable within IDDSI level over a timeframe of 3 h.

### Temperature Testing

Exploration of the impact of temperature was conducted using barium thickened with 0.35, 0.5, 1.0, 2.0, and 4.2 g/100 ml of xanthan gum and with 3, 3.3, 3.9, 5.1, and 6.7 g/100 ml of starch. The initial batch was divided into portions that were maintained either at room temperature or stored in a refrigerator (at approximately 4 degrees Celsius). Repeated flow tests were performed on both room-temperature and chilled samples at 1-, 2-, and 3-h post mixing.

## Analyses

Means and ranges for flow test results were calculated by thickener concentration (g/100 ml) for each timepoint of measurement. Descriptive statistics and graphs will be used to illustrate the results of the flow testing.

For the demonstration of recipe stability, we conducted repeated measures ANOVAs of IDDSI Flow Test result within liquid type (barium/non-barium plus starch or gum) across time with a covariate of concentration. Similarly, for the exploration of temperature-based variations, we conducted a repeated measures ANOVA of IDDSI Flow Test result within liquid type (starch; gum) for matched chilled versus room-temperature barium recipes across timepoints with a covariate of concentration.

## Results

**Fig. 1:**
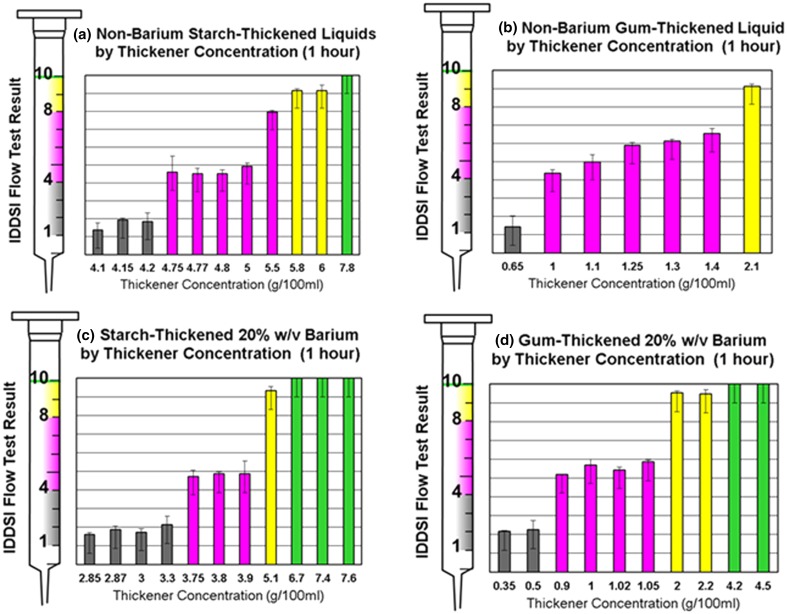
IDDSI Flow Test results at 1 h for **a** non-barium starch, **b** non-barium xanthan gum, **c** starch-thickened barium, and **d** xanthan-gum-thickened barium at 1-h post mixing

Trends in the flow test results across thickener concentrations at the 1-h timepoint post mixing can be seen in Fig. 1a–d. A clear pattern of higher flow test residual volumes (i.e., thicker liquids) is seen with higher concentrations of thickener within each liquid type. The stability analysis showed this result to be statistically significant for all four liquids, with *p*-values ranging from < 0.001 to 0.002.

Across time, none of the recipes showed a shift across IDDSI level boundaries. However, statistically significant increases in thickness within IDDSI level were found in pairwise comparisons between the 1- and 2-h (*p* = 0.009) and the 2- and 3-h timepoints (*p* = 0.004) for the non-barium starch-thickened stimuli. This effect was not found for the other liquids.

**Fig. 2:**
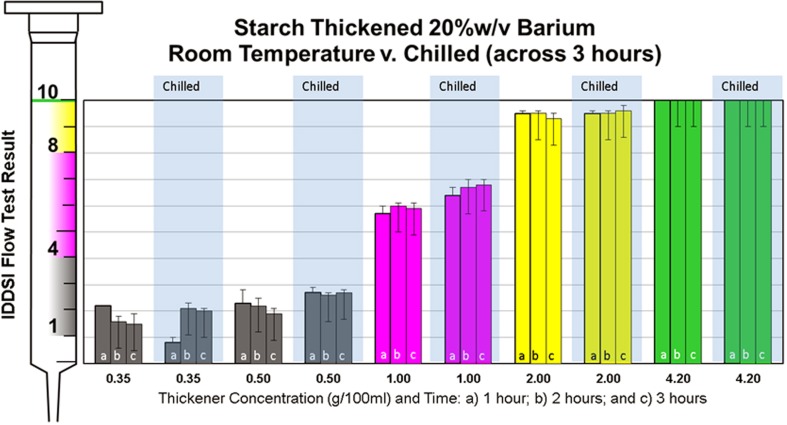
IDDSI Flow Test results for room-temperature and chilled starch-thickened barium over 3 h

Figures 2 (starch) and 3 (gum) illustrate differences in flow of thickened barium between room-temperature and refrigerated samples across time. Although the refrigerated barium stimuli were thicker than the room-temperature stimuli, this difference was not statistically significant.

**Fig. 3:**
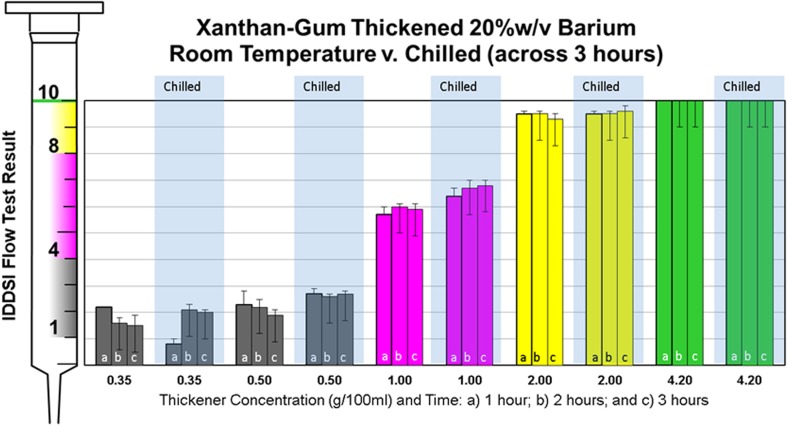
IDDSI Flow Test results for room-temperature and chilled xanthan-gum-thickened barium over 3 h

## Discussion

The creation of assessment stimuli that have similar flow properties to non-barium liquids that are available for dysphagia management is important for patient care and diagnosis. The IDDSI Flow Test provides clinicians with a means of confirming similarity in flow across liquids. This study was designed to identify recipes for slightly, mildly, and moderately thick liquid barium stimuli that would remain stable at 1 h after mixing and up to 3 h afterward. Final recipes of the barium and non-barium stimuli can be found in Table 1. The results underscore the importance of clinicians developing awareness of factors that can contribute to variations in the flow of liquids and of methods for measuring flow. This is of particular relevance when preparing barium stimuli for use in videofluoroscopy.

**Table 1:** Final recipes (g/100 ml) for all non-barium and barium liquids by IDDSI level

IDDSI level	Non-barium		Barium	
	Xanthan gum	Starch	Xanthan gum	Starch
1	0.65	4.15	0.4	2.85
2	1.25	4.77	1.02	3.75
3	2.1	5.85	2.2	5.1

The study highlights the flow of four different types of liquid, depending on thickener, and whether or not barium has been added. Figure 1a–d shows the decrease in flow that was seen as the amount of thickener was increased. It is important to highlight that the amount of thickener needed to reach any given level of thickness on the IDDSI continuum is much lower when using xanthan-gum- rather than starch-based thickeners. For example, Fig. 1a and b show non-barium liquids thickened with the starch and xanthan-gum thickeners, respectively. In both graphs, the first data point shows a slightly thick liquid with an average of 1.4 ml left in the syringe after 10 s of flow. For the starch-thickened liquid (Fig. 1a), 4.1 g/100 ml of thickener was required to reach this level; the corresponding xanthan-gum-thickened liquid (Fig. 1b) required only 0.65 g/100 ml of thickener to achieve the same flow. The common trend for thickener labels to specify the amount of thickener needed using a simplified 1-, 2-, or 3-scoop methodology obscures this difference. Users may not be aware that the size of the scoop included in a package of thickener has been adjusted to compensate for the different concentrations required across thickener types.

Several of the recipes that were tested in this study yielded IDDSI Flow Test results of 10 ml (i.e., no drip). It is important to mention that this result represents saturation of the IDDSI Flow Test. Confirmation that a liquid falls in the IDDSI extremely thick liquid category requires supplementary spoon tilt and fork drip tests as per IDDSI guidelines. It is acknowledged that these supplementary tests are subjective and do not permit precise matching of flow characteristics across liquids within this level.

In some cases, the recipes in this study produced flow test results that fell close to the boundaries of the IDDSI levels. For example, in Fig. 1b, 1.0 g/100 ml of xanthan-gum thickener added to water produced a liquid with an average flow test result of 4.5 ml, just above the lower boundary of the IDDSI mildly thick range. In such cases, our data show that small changes in thickener concentration can shift the liquid closer to the center of the target IDDSI range. In the example cited, the addition of 0.3 g of xanthan gum (1.3 g/100 ml) moved the flow test result to the middle of the mildly thick range at 6.1 ml. Until clearer evidence regarding the clinical significance of small differences in liquid flow is available [2] we recommend that clinicians target flow test results closer to the middle of each IDDSI range.

The reconstitution of barium sulfate powders with water and thickening agents is a widespread but off-label practice that has arisen due to restricted access to pre-thickened barium products outside the United States. The variations in flow that were seen across small differences in thickener concentration in this study underscore the need to follow recipes when preparing barium for videofluoroscopy. Clinicians should not assume that addition of the same amount of thickener to a non-barium liquid and a barium suspension will result in liquids with similar flow. For example, in Fig. 1b 1.0 g/100 ml of xanthan-gum thickener added to water resulted in an average flow test result of 4.4 ml at the 1-h timepoint post mixing; by contrast, Fig. 1d shows that the same amount of xanthan-gum thickener (1.0 g/100 ml) in a 20% w/v barium suspension resulted in a thicker liquid with a flow test result of 5.7 ml. Despite this difference, both of these results fall in the IDDSI mildly thick range; it is not yet known whether a flow difference of this magnitude has clinical significance. However, more dramatic differences are seen with the starch-based thickener. As seen in Fig. 1a, 5 g/100 ml of starch-based thickener in water yields a mildly thick liquid with a flow test result of 4.9 ml. However, a similar amount of the same thickener (Fig. 1c; 5.1 g/100 ml) added to a 20% w/v barium suspension resulted in a moderately thick liquid with a flow test result of 9.3 ml. These examples are consistent with previous literature [21–23] showing that the combination of barium and thickening agents may alter multiple rheological parameters, and specifically may lead to further thickening. In addition, it must be emphasized that the barium stimuli in this study were prepared in a low concentration (20% w/v); one might expect that the addition of thickener to higher concentrations of barium may lead to even greater thickening. The reader should be cautioned that the interactions illustrated in this study are likely to be specific to the particular brands of barium suspension and/or thickener studied, based on their composition, and the patterns seen cannot be generalized to other products.

When comparing room-temperature liquids to liquids that were chilled, we found very few differences in flow. All of the barium-based recipes yielded stable IDDSI Flow Test results over time, up to 3 h. The addition of refrigeration as a variable had very little impact. As seen in Fig. 2, only one barium-thickened liquid recipe (3.3 g/100 ml starch) showed temperature-related thickening which crossed the boundary from slightly to mildly thick for the chilled product.

## Limitations

This study is not without limitations. The thickeners tested are limited to two particular products, mixed with a single commercially available barium sulfate suspension. The results may not be generalizable to other products. Furthermore, these products were reconstituted with commercially available bottled waters. In clinical practice, off-label use of barium may involve reconstitution with other liquids, such as infant formula or breastmilk [24, 25]. Clinicians should be cautioned that the kinds of interactions seen between thickeners and barium in the current study may also occur when thickeners or barium is added to other liquids, based on the protein or macronutrient composition of the liquid [16]. In such cases, the IDDSI Flow Test provides a means of checking flow and confirming similarity between the barium and non-barium product.

## Conclusions

A practical goal of this study was to determine recipes for matched non-barium and barium stimuli, which would fall clearly within the slightly to moderately thick levels of the IDDSI framework and remain in those levels over a 3-h timeframe post mixing at room temperature. This goal was achieved. As a rule, the combination of barium powder and thickener led to additional thickening compared to thickened liquids prepared without barium. Adjustments to the thickener amounts were required to correct for this interaction, particularly when using starch-based thickeners.
